# Placebos in clinical care: a suggestion beyond the evidence

**DOI:** 10.5694/mja2.51230

**Published:** 2021-09-19

**Authors:** Christopher G Maher, Adrian C Traeger, Christina Abdel Shaheed, Mary O'Keeffe

**Affiliations:** ^1^ University of Sydney Sydney NSW; ^2^ Institute for Musculoskeletal Health University of Sydney Sydney NSW

**Keywords:** Placebos

The recent enthusiasm for the clinical use of placebos seems driven by myths and misunderstandings

In the past few years, major medical journals have published commentaries considering whether placebos may have a role in clinical care. Some commentators seem to have concluded that placebos should be part of clinical care and have provided advice on implementation: “Clinician education, training manuals, and workshops might help with initial implementation”.^1^

The clinical use of placebos is often justified by pointing to their important treatment effects. Proponents have also argued that the placebo effect can be amplified by manipulating the physical appearance of the pill,^2^ that placebo effects vary across geographic regions,^3^ and that placebos are getting more effective over time.^4^, ^5^ It has even been suggested that subterfuge is unnecessary; placebos can be honestly described as inert and still yield important clinical effects.^1^, ^6^ Given the recent advocacy for the clinical use of placebos, it is timely to consider the evidence underpinning these claims.

## Placebos provide important treatment effects

Placebos are not new. A 1752 treatise advocated placebos for women in labour to hasten delivery.^7^ Over time, placebos somehow gained the reputation of remarkable therapeutic effectiveness; well illustrated by the title of Beecher’s highly cited 1955 review *The powerful placebo*.^8^ However, in Beecher’s review, the placebo results were quite modest. For example, in one of his trials cited in the review,^8^ all of the nine soldiers given methadone reported satisfactory pain relief, whereas only two of the nine given a placebo reported satisfactory pain relief. The modest outcomes in the placebo group are even less impressive as they include improvement due to factors other than placebo, such as the natural course of the condition. The key limitation of Beecher’s work is that his measure of the placebo effect was within‐group change in the placebo group. This effect estimate is biased and means that Beecher substantially overestimated the size of the placebo effect.

Reviews that appropriately estimate the placebo effect as the difference in outcomes between placebo and no treatment groups suggest that placebos usually have modest effects. A Cochrane review of placebos^9^ considered 234 trials and concluded that, in general, placebos do not produce major health benefits, except for some small and inconsistent effects on self‐reported outcomes such as pain or nausea. For example, there was no effect on pain measured using a dichotomous scale (risk ratio, 0.92; 95% CI, 0.77–1.11; 6 trials, *n* = 1207) and a small effect if pain was measured using a continuous scale (standardised mean difference [SMD], 0.28; 95% CI, 0.19–0.36; 60 trials, *n* = 4154). The continuous effect size equates to about five units on a zero to 100 pain scale, an effect quantifiable in a large review but unlikely to be discerned by a typical patient. This suggests limited clinical utility.

## The physical appearance of a placebo pill influences the size of the placebo effect

The colour, size and shape of a placebo pill have all been suggested to influence its effect.^2^ However, this view does not have a firm basis in science. A key problem is that in many studies evaluating the influence of the appearance of a placebo pill, the participants did not consume the pill or have a health condition. One review^2^ considering placebo features located nine studies of healthy people (*n* = 1294) testing the effect of the colour of a placebo pill or capsule and only three studies of patients (*n* = 166). A common design is that researchers have asked healthy people which pill they think would work best; often not even specifying an outcome or health condition. For example, one study asked 20 young healthy men and women to rank the potency of white, black, blue, red, green, orange and yellow capsules on a scale from 1 (strongest) to 7 (weakest).^10^ The effect of the physical appearance of a placebo pill has been tested in a few clinical studies (ie, where patients consumed the placebo pill), but the studies are small and have yielded inconsistent results. For example, one study commonly cited to show superiority of red placebos enrolled 22 subjects, with five subjects taking red placebos.^11^ The notion that placebo pill appearance is important is based on a very small and weak evidence base.

## Placebos, even when honestly described as inert, create important clinical effects

It has been recently suggested that it may be possible for clinicians to openly describe placebos as inert, but still elicit important clinical effects.^1^ So‐called open label placebos have moved beyond research settings and it is now possible to purchase Zeebo (Zeebo Effect) placebo pills branded as “Zeebo Relief Pure Honest Placebo”. The claims for open label placebo pills are becoming more surprising, with one recent trial reporting that the beneficial effects of a 3‐week course of an open label placebo pill lasted for 5 years in patients with chronic low back pain.^12^ But much of the enthusiasm for open label placebos is based on doubtful science. The trial^12^ that claimed 5‐year beneficial effects of an open label placebo, for example, followed up the participants who received the open label placebo but not the control participants who did not receive the placebo. This practice converted the randomised controlled trial into a case series and created considerable uncertainty about what is the true long term effect of an open label placebo.

Research on open label placebo has some limitations. A review^13^ of open label placebos highlighted the weak evidence base and concluded that it was premature to consider their use in clinical practice. A key concern is that the typical study design biases outcomes. All subjects are extensively briefed on why placebos work, but then those in the control group are told that they will not receive a placebo. The briefings are so extensive that clinical applicability is doubtful. In one trial,^6^ the initial briefing lasted 15 minutes and was repeated at day 11. Given that in most parts of the world the length of a primary care consultation is 5 minutes or less,^14^ the briefing required to prime patients would not be feasible. It is also arguable that with such extensive briefings the placebo is not being honestly portrayed as inert.

A puzzling pattern is that open label placebo trials typically report much greater effects than trials of closed placebos. For example, a review^15^ of open label placebo trials (13 trials, *n* = 834) reported an SMD of 0.72, which is three times larger than the effect of a closed label placebo trial reported in a Cochrane review^9^ (202 trials, *n* = 16 566). This result makes little sense, given that placebo effects are thought to be mediated by expectancies. The Cochrane review investigated the effect of expectancies and found that placebo effects were twice as large in trials where patients were incorrectly told that there was no placebo arm (SMD, 0.39), compared with trials where patients were correctly informed about the possibility of receiving a placebo (SMD, 0.19). It is hard to understand how patients in open label placebo trials, who are told they have no chance of receiving an active substance, would do better than patients in closed label placebo trials, who are told they have some chance of receiving an active substance.

## Placebos are getting more effective over time; different geographical locations produce different placebo effects

These placebo facts were presented in recent editorials,^3^, ^4^ but the studies cited as evidence did not measure the placebo effect. A review of treatment for dementia, for example, incorrectly calculated the placebo effect as within group change in the placebo arm.^5^ But there is a bigger issue: it is impossible to manipulate time, and challenging to manipulate geographic location, within a randomised trial. The evidence that these two factors modify the effect of placebo could only come from non‐randomised comparisons; and confounding would be a major concern. Even if we ignored these problems, we do not see how these two placebo facts could help a clinician administer a placebo with greater therapeutic effect. It is an example of how much of the current discourse on placebo seems to focus more on enshrining placebos as mysterious and highly effective and less on making a practical difference to patient care and outcomes.

## Conclusion

Placebos remain important for clinical trials because they help achieve blinding and, thus, control of bias; however, there is no evidence that placebos have much to offer for clinical care. When administered in a blinded fashion, a placebo will provide a small effect, but the real treatment will normally provide better outcomes for the patient. A placebo supporter could counter that they could get a bigger placebo treatment effect by dispensing large, red, unusually shaped placebo pills, but they would be basing this on uncertain evidence. It may be better to dismiss placebos and instead manage patients with evidence‐based treatments.

## Competing interests

No relevant disclosures.

## Provenance

Not commissioned; externally peer reviewed.
